# Prognostic factors for mortality among patients with visceral leishmaniasis in East Africa: Systematic review and meta-analysis

**DOI:** 10.1371/journal.pntd.0008319

**Published:** 2020-05-15

**Authors:** Charles Abongomera, Saskia van Henten, Florian Vogt, Jozefien Buyze, Kristien Verdonck, Johan van Griensven

**Affiliations:** 1 Médecins Sans Frontières, Abdurafi, Ethiopia; 2 Department of Clinical Sciences, Institute of Tropical Medicine, Antwerp, Belgium; 3 Department of Public Health, Institute of Tropical Medicine, Antwerp, Belgium; Universidade do Estado do Rio de Janeiro, BRAZIL

## Abstract

**Background:**

Visceral leishmaniasis (VL) is a vector-borne disease that is deadly if left untreated. Understanding which factors have prognostic value may help to focus clinical management and reduce case fatality. However, information about prognostic factors is scattered and conflicting. We conducted a systematic review and meta-analysis to identify prognostic factors for mortality among VL patients in East Africa.

**Methodology/Principal findings:**

The review protocol was registered in PROSPERO (CRD42016043112). We included studies published in English after 1970 describing VL patients treated in East African health facilities. To be included, studies had to report on associations between clinical or laboratory factors and mortality during admission or during VL treatment, with a minimal study size of ten patients. Conference abstracts and evaluations of genetic or immunological prognostic factors were excluded. We searched for studies in MEDLINE and four other databases in December 2018. To assess the risk of bias in observational studies and clinical trials, we used the Quality in Prognostic Studies (QUIPS) tool. We included 48 studies in the systematic review, describing 150,072 VL patients of whom 7,847 (5.2%) died. Twelve prognostic factors were evaluated in five or more studies and these results were submitted to meta-analysis producing one pooled crude odds ratio (OR) per prognostic factor. The following factors were strongly (OR>3) and significantly (P-value<0.05) associated with mortality: jaundice (OR = 8.27), HIV (OR = 4.60), tuberculosis (OR = 4.06), age >45 years (OR = 3.69), oedema (OR = 3.52), bleeding (OR = 3.37), and haemoglobin ≤6.5 g/dl (OR = 3.26). Factors significantly and moderately (OR between one and three) associated with death were severe malnutrition, long duration of illness, young age (<5 years), and large spleen size.

**Conclusions/Significance:**

These prognostic factors can be identified by health professionals in resource-constrained settings. They should be considered as “core” prognostic factors in future studies that aim at improving the prognosis of VL patients.

## Author summary

Visceral leishmaniasis (VL) is a severe disease caused by a micro-organism called *Leishmania*. This disease occurs in South America, East Africa, the Mediterranean, and Asia. There are several treatments with differing success rates, side effects, and costs. Some of the VL patients who seek care in health facilities die, and knowing which patients are at risk of dying is useful for patient management. Indeed, it makes sense to reserve the best treatment and closest follow-up for those patients with the highest risk of dying. We searched the medical literature in December 2018 and found 48 studies evaluating associations between clinical or laboratory factors and risk of dying among VL patients treated in East Africa. These studies described 150,072 VL patients of whom 7,847 (5.2%) died. Factors that were evaluated in five or more studies were analysed further. VL patients were at increased risk of dying if they had jaundice, HIV, tuberculosis, old (more than 45 years) or young (below five years) age, oedema, bleeding, anaemia, malnutrition, long duration of illness, and large spleen size. We recommend to include these factors, which can easily be detected by health professionals, in future studies that aim at improving the prognosis of VL patients.

## Introduction

Visceral leishmaniasis (VL), or kala-azar, is a neglected tropical disease which is deadly if untreated [1,2]. In East Africa and the Indian subcontinent, it is caused by *Leishmania donovani* and in Latin America and the Mediterranean by *L*. *infantum*. *Leishmania* parasites are transmitted through the bite of infected female phlebotomine sandflies [1,2]. The cycle of *L*. *donovani* is predominantly anthroponotic and that of *L*. *infantum* usually includes dogs or other mammals [1,2]. VL is endemic in 75 countries, and the estimated annual global incidence is 50,000–90,000 new cases [3–5]. In 2015, East Africa contributed the highest proportion of new VL cases world-wide– 40% [3]. South Sudan and Sudan have the highest case load, followed by Ethiopia [6–8]. VL has also been reported in Kenya, Uganda, Eritrea and Somalia [3,9–11].

VL patients usually present with prolonged fever, weight loss and splenomegaly [1,2,12]. Sodium stibogluconate (SSG), the main VL treatment in East Africa, may cause potentially lethal adverse events such as cardiac arrhythmia [13,14]. Liposomal amphotericin B (AmBisome), the alternative drug, is safer but expensive–therefore it should be administered to patients who need it most, such as those with severe VL or those at risk of adverse events with SSG [15]. The average VL case fatality rate in East Africa was 2% in 2015, but this proportion can be markedly higher in specific subgroups, such as in VL and HIV co-infected patients among whom the case fatality rate can be as high as 39% [16,17].

A critical step to improve patient outcomes is a deeper understanding of the factors that determine the prognosis among patients with VL. Evidence-based clinical decision tools based on key prognostic factors are increasingly used in other clinical domains to identify high-risk patients requiring specific care such as more intensive monitoring, additional investigations or specific treatments [18,19]. On the other hand, patients with a better prognosis might be treated on ambulatory basis or at a decentralised level [18,19]. Similarly, the stratification of patients with VL according to their risk of death could help to focus clinical management and reduce case fatality.

A systematic review reporting on prognostic factors for mortality has been reported from Latin America, where VL is caused by *L*. *infantum* [20]. A wide range of prognostic factors were found to be associated with mortality ranging from clinical signs and symptoms (jaundice, oedema, ascites, bleeding, pronounced splenomegaly, vomiting, and diarrhoea) to laboratory abnormalities (anaemia, thrombocytopenia) and coinfections (HIV and tuberculosis) [20]. However, there are important differences in reports from different geographical regions regarding many aspects related to VL, such as clinical presentation, disease severity, case fatality and treatment response [12,15]. Whether this relates to differences in *Leishmania* species, host population, health-seeking behaviour, or style of reporting remains largely unexplored. Consequently, whether prognostic factors identified in Latin America also apply to East Africa where *L*. *donovani* is prevalent is unknown.

Although several studies in East Africa have aimed at identifying prognostic factors [21–26], the information is scattered and sometimes conflicting, and it is currently unclear to which extent the findings of these studies are valid and generalizable. Hence, the classification of VL severity remains poorly defined. We therefore set out to conduct a systematic review to identify key prognostic factors associated with mortality among patients with VL in East Africa.

## Methods

### Protocol and registration

This review was conducted in line with the ‘preferred reporting items for systematic reviews and meta-analyses’ (PRISMA) guidelines [27]. The completed PRISMA checklist is available in S1 Text. The review protocol was registered in a repository of systematic review protocols prior to starting the research (PROSPERO, protocol number CRD42016043112) [28].

### Eligibility criteria

We aimed to include studies on VL patients from East African countries, namely Ethiopia, Sudan, South Sudan, Kenya, Uganda, Somalia and Eritrea, with a laboratory-confirmed or clinical VL diagnosis and treated in hospitals, health centres or other health care structures. Studies had to evaluate the association between clinical or laboratory parameters and mortality during admission or during VL treatment, with a minimal study size of ten patients. To be included, studies had to provide either a summary measure for the association with mortality [odds ratio (OR), relative risk (RR), or hazard ratio (HR)] with a P-value or confidence interval (CI), or had to give crude data that allowed the calculation of a measure of association. We excluded conference abstracts and studies on prognostic factors relating to genetic or immunological features. Only studies published after 1970 and in English language were considered.

### Information sources, search strategy, and study selection

In December 2018, we searched for studies in five electronic databases. The search strategy combined terms indicating the disease (such as “kala azar” and “visceral leishmaniasis”) with terms indicating the geographical region. Details are given in Table 1.

**Table 1:** Search terms and date of search in the different electronic databases.

Database	Search Terms	Date of search
PubMed/Medline	((((((("visceral leishmaniasis") OR "kala azar") OR kala-azar) OR "leishmaniasis, visceral"[MeSH Terms])) AND (("east africa") OR east-africa OR Ethiopia* OR Sudan* OR Kenya* OR Uganda* OR Somalia* OR Eritrea* OR "africa, eastern"[MeSH Terms])))	7/12/ 2018
Google scholar	• "visceral leishmaniasis" ((ethiopia OR sudan OR eritrea OR kenya OR uganda OR somalia OR East-Africa OR "East-Africa" OR "Eastern Africa") • kala-azar ((ethiopia OR sudan OR eritrea OR kenya OR uganda OR somalia OR East-Africa OR "East-Africa" OR "Eastern Africa")	7/12/2018
The Cochrane Library (Cochrane Database of Systematic Reviews, Cochrane Central Register of Controlled Trials (CENTRAL), Cochrane Methodology Register)	# 1 "visceral leishmaniasis" or "kala azar" or kala-azar # 2 MeSH descriptor: [Leishmaniasis, Visceral] explore all trees #3 “east africa” #4 MeSH descriptor: [Africa, Eastern] explore all trees #5 (#1 or #2) and (#3 and #4)	7/12/2018
ClinicalTrials.gov	East africa & Visceral Leishmaniasis	7/12/2018
World Health Organization (WHO) International Clinical Trials Registry Platform (ICTRP).	Visceral Leishmaniasis AND Eastern-Africa	7/12/2018

Additionally, we reviewed the reference lists of selected publications and contacted VL specialists to check if the automatic search had missed any important studies.

Titles and abstracts of all retrieved studies were independently screened by two authors (CA and SvH). These same authors also independently assessed the full texts of the retained studies. At both steps, disagreements were resolved by a third author (FV).

### Data items and data collection process

The core information was the strength of association between prognostic factors and mortality. In addition, we extracted information about study setting (place, time, type of health facility); study population (demographics, clinical characteristics, inclusion/exclusion criteria); type of treatment; and study design and methods (including information needed for the risk of bias assessment). Data from the included studies were extracted using a standardised, pre-piloted form. Two authors (CA and SvH), independently extracted the data. Disagreements were resolved by a third author (JvG).

### Risk of bias in individual studies

Two authors (CA and SvH) independently assessed the risk of bias in the studies using the Quality in Prognostic Studies (QUIPS) tool [29]. Disagreements were resolved by a third author (JvG/FV). The results of the risk of bias assessment are described as part of the narrative synthesis but were not used in the meta-analysis.

### Synthesis of results

All included studies are presented in a narrative synthesis and summary table. For each prognostic factor evaluated in at least three studies, we also summarised the findings in a forest plot. For factors reported in at least five studies, we did a meta-analysis. The information that was pooled was the strength of the association between a specific prognostic factor and mortality during admission or VL treatment. The summary measure was a pooled OR for each candidate prognostic factor.

As different studies reported continuous variables with different cut-offs, we defined standardised cut-offs based on information from the literature and the available data. For age we used <5, <15, 15–45 and ≥45 years, for duration of illness <2 months and ≥2 months, for malnutrition body mass index (BMI) <16 kilogrammes/metre^2^ (kg/m^2^) or Z score <-3, for haemoglobin ≤6.5 grams/decilitre (g/dl) and >6.5 g/dl, and for spleen size <10 centimetres (cm) or ≥10 cm.

Current guidelines for prognostic studies recommend to report both crude and adjusted measures of association [30]. However, adjusted measures are comparable only if they are based on multivariable models that include a comparable set of variables (key or core prognostic factors). As most of the studies included in our review either did not report adjusted measures or presented findings based on differing sets of variables, we decided to use only the crude measures of association for our meta-analyses. Findings from the few studies reporting multivariable analyses were described separately in the narrative synthesis. For these studies, we also reported on the types of models used, the procedures used for building the models, how validation were conducted and how performance of the models were assessed.

For the different predictors, we performed a fixed effects and random effects meta-analysis of the odds ratio of mortality. Since we pooled studies conducted in different settings and with high heterogeneity, the main conclusions were based on the random effects model. However, we also reported the fixed effects model for comparison. The amount of heterogeneity was quantified with the I square statistic (*I*^*2*^), which expressed the proportion of variation across studies that is due to heterogeneity.

We performed subgroup analyses for prognostic factors that were evaluated in at least five studies and where a variable of interest for subgroup analysis was also reported. Therefore, subgroup analyses for some prognostic factors were not conducted as they were studied in less than five studies. Subsequently, we only conducted subgroup analyses according to the HIV prevalence and the countries where the studies were conducted. The HIV prevalence was stratified as follows: HIV prevalence >90%, 10–90%, <10%, and unknown HIV prevalence.

For all prognostic factors evaluated in meta-analyses, funnel plots were made to assess the risk of publication bias [31,32]. Funnel plots visualize for all the included studies their precision against the reported strength of association. Plots taking the shape of a symmetric, inverted funnel suggest that publication bias is unlikely. All statistical analyses were performed with R version 3.6.0.

## Results

### Characteristics of the selected studies

**Fig 1:**
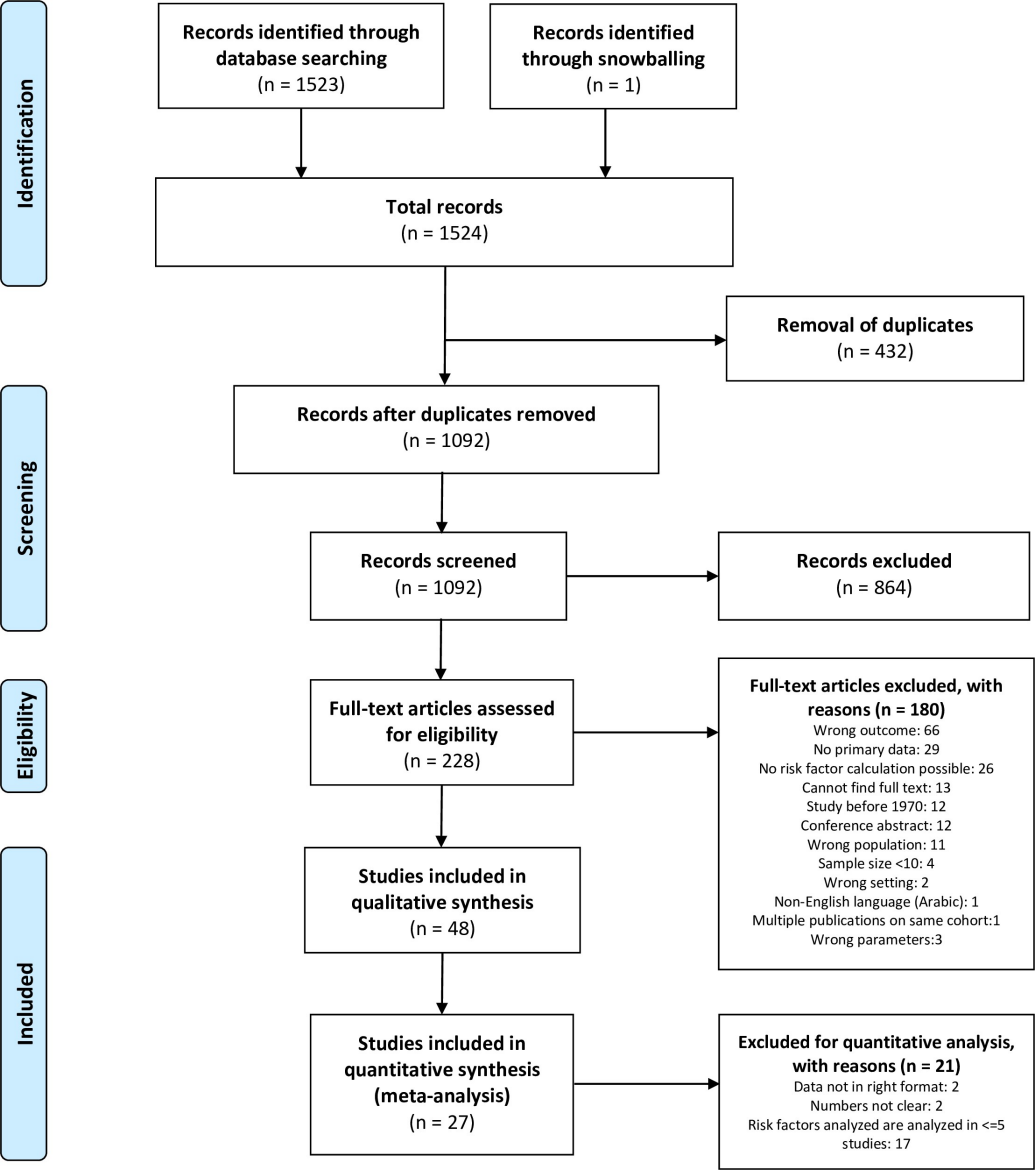
Flow diagram of the studies identified, screened, reviewed, and included in the systematic review and meta-analysis.

Of the 1,524 studies identified, 48 were included in the systematic review [17,21–26,33–73] (Fig 1).

Most studies included in the systematic review were observational [77.0%, (37/48)] and the rest were clinical trials [23.0%, (11/48)] (S1 Table and S2 Table). Of the 11 trials, eight were randomised and three non-randomised. Most studies were conducted in Ethiopia [33.3%, (16/48)], followed by Sudan [31.3%, (15/48)], South Sudan [12.6%, (6/48)], Uganda [6.3%, (3/48)], Kenya [6.3%, (3/48)], and the rest were multicentre studies [10.4%, (5/48)]. Approximately one third (17/48, 35.4%) of the studies were conducted through a collaboration of non-governmental and governmental institutions, while 14 (29.2%) were conducted by non-governmental institutions and 13 (27.1%) by governmental institutions. Most studies [32/48 (66.6%)] were conducted in hospitals (S1 Table and S2 Table).

Patient inclusion criteria varied across studies: some studies included all VL patients, whereas others had strict enrolment criteria. For example, some studies focused on specific VL populations, such as HIV coinfected patients [36,45,57,72], people with severe VL or those failing first line treatment [45,62], patients on specific VL treatment regimens [23,36,40,46,52,56,72], or pregnant women [33]. There were also studies that excluded patients with the above criteria (e.g. HIV coinfected patients [35,39,56,69], or pregnancy [35,37,39,64,69]).

The total number of patients in the 48 studies [17,21–26,33–73] included in the systematic review was 150,072 and 7,847 (5.2%) of them died (S1 Table and S2 Table). In the observational studies [17,21–26,33–59,71–73], there were 146,698 patients and 7,703 (5.3%) died (S1 Table), whereas in the trials [60–70], there were 3,374 patients and 144 (4.3%) died (S2 Table). Patients from Sudan made up 72.2% of the total number, since most of the studies conducted there were large, while only 4.7% of all patients were from Ethiopia, where many studies were of smaller size. There were no studies included from Eritrea or Somalia (S1 Table and S2 Table). Out of the 48 studies [17,21–26,33–73], 27 were included in the meta-analysis [17,21–26,33–47,60,65,71–73] (Fig 1).

Pentavalent antimonials were the most commonly used treatment regimen. They were used in 39/48 studies and the death rate ranged from 0 to 55.6% (mean 6.8%) [17,21–26,34–42,47–55,57–66,68,70,71,73]. Liposomal amphotericin based regimens were used in 19/48 studies and the death rate ranged from 0 to 12.7% (mean 7.9%) [21,24,34,38,39,44–46,48,53,54,56,57,62,67,69,71–73].

### Risk of bias

**Fig 2:**
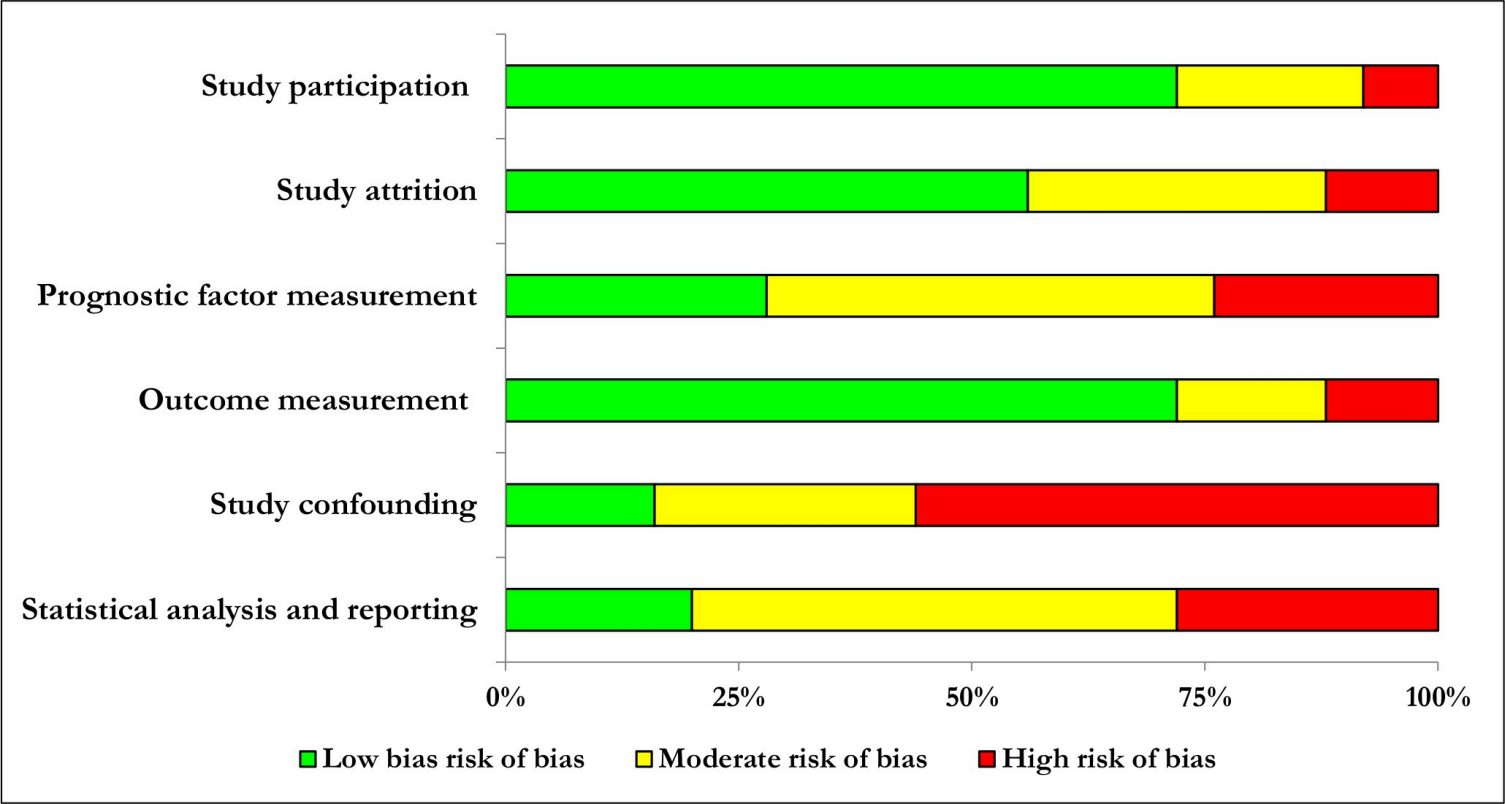
Risk of bias assessment according to the six domains of the Quality in Prognostic Studies (QUIPS) tool for 25 observational studies included in the meta-analysis.

The risk of bias assessment for all the observational studies included in the systematic review (n = 37) is given in S1 Fig, and the assessment for the observational studies included in the meta-analysis (n = 25) in Fig 2.

The majority of these 25 studies [17,21–26,33–47,71–73] scored well (low risk of bias) in three of the six domains of the QUIPS tool, i.e. study participation, study attrition, and outcome measurement. The other three domains, i.e. prognostic factor measurement, study confounding, and statistical analysis and reporting were more problematic. Here, the majority of studies were classified as having moderate or high risk of bias (Fig 2).

The bias assessment for the 11 trials [60–70] included in the systematic review is given in S2 Fig. The two trials [60,65] included in the meta-analysis scored relatively well. In one trial [65], the risk of bias was low in all the domains except in study confounding, where the risk of bias was moderate. In the other trial [60], the risk of bias was low in the outcome measurement and study confounding domains, but it was moderate in all the other domains (S2 Fig).

### Prognostic factors for mortality

A wide variety of potential prognostic factors were evaluated in the included studies (Table 2).

**Table 2:** Overview of all prognostic factors evaluated.

Prognostic factors	Number of studies	N significant^a^/N that checked significance^b^	References of N significant
**Socio-demographic**			
- Age	17	11^c^/14	[17,22–26,38,48,55,60,72]
- Female sex	8	1/7	[23]
- Rural residence	2	2/2	[33,73]
- Education	1	0/1	
- Literacy	1	0/1	
- Parity	1	0/1	
- “Black race”	1	1/1	[33]
**Clinical symptoms**			
- Malnutrition	13	7/11	[17,22,24,25,38,55,60]
- Spleen size	8	2^d^/7	[22,38]
- Bleeding	7	4/6	[17,22,25,55]
- Diarrhoea	6	4/5	[22,23,25,60]
- Vomiting	6	6/6	[22,24–26,55,60]
- Jaundice^e^	5	3/4	[23,25,38]
- Oedema^f^	5	2/4	[24,38]
- Weakness	5	3/4	[25,38,60]
- Pregnancy	2	1/1	[23]
- Ascites	1	0/0	
- Lymphadenopathy	1	1^g^/1	[38]
- Darkened skin	1	1/1	[17]
- Neurological complications	1	1/1	[25]
- Adverse events	1	1/1	[23]
**HIV related**			
- HIV infection	11	5/6	[17,24,25,44,60]
- CD4 count	2^h^	0/2	
- ART use	1	0/1	
- Concurrent VL-HIV diagnosis	1	0/0	
- Early ART initiation	1	1/1	[34]
**Coinfections**			
- Tuberculosis	5	2/3	[23,24]
- Malaria	4	1^i^/3	[59]
- Acute respiratory infection	1	0/1	
- Ear, nose or throat infection	1	0/1	
- Hepatitis B	1	0/0	
- Hepatitis C	1	0/0	
- HIV/Hepatitis B/Hepatitis C/Malaria	1	0/0	
- Pneumonia	1	1/1	[24]
**Laboratory tests**			
- Low haemoglobin	9	7/8	[22,24,26,38,55,60,72]
- Low haematocrit	2	1/1	[17]
- High parasite load	3	2/3	[72,74]
- Low white blood cells	1	0/0	
- Low lymphocytes	1	0/0	
- Low platelets	1	0/0	
- Albuminuria	1	0/0	
- Serum protein	1	0/0	
- Positive formal gel test	1	0/0	
**Health seeking behaviour**			
- Long duration of illness	10	5/9	[22,26,33,48,55]
- Attendance of prenatal care	1	0/1	
- Long distance to the hospital	1	0/1	
**Relapse VL**	9	1^j^ /5^k^	[72]
**VL treatment regimen**	24	3/8	[48,52,60]

Abbreviations: ART, antiretroviral therapy; HIV, human immunodeficiency virus; VL, visceral leishmaniasis.

^a^ Studies that reported a significant association in text, a P-value <0.05, or 95% confidence intervals not including one.

^b^ Either studies that reported on association in text, P-values or 95% confidence intervals.

^c^ Both old age and young age are associated with death.

^d^ Only significant in children in one paper, only significant in adults in the other paper.

^e^ Includes one paper that assessed liver disease (clinical jaundice and ⁄or a positive hepatitis B surface antigen).

^f^ Some papers combined oedema and/or ascites.

^g^ Only significant in children.

^h^ One paper assessed CD4<50 and/or WHO stage IV together.

^i^ In one paper 2 cohorts are included, malaria increased chance of death in one cohort, not in the other.

^j^ Primary VL has a higher chance of death in HIV patients.

^k^ The findings from Boateng *et*. *al*. [48] were not interpreted as being significant since PKDL was used as the reference for two separate comparisons (relapse VL *vs*. PKDL and primary VL *vs*. PKDL, rather than primary VL *vs*. relapse VL).

The prognostic factors that were assessed most frequently (in at least ten studies) were age, malnutrition, HIV status, duration of illness and treatment regimen. On the other hand, many factors were assessed in only one or two studies (Table 2).

The funnel plots for 12 prognostic factors are shown in supplementary information (S2 Text). The number of observations on the plots (i.e. the number of studies per prognostic factor) was relatively low (ranging between 5 and 11). The funnel plots that were least symmetrical were those for age and relapse VL, but overall, there were no indications of substantial publication bias.

### Meta-analysis

Twelve prognostic factors (reported in 27 studies) were evaluated in at least five studies and were submitted to meta-analysis. The overview of the meta-analyses of the crude ORs can be found in Table 3.

**Table 3:** Overview of prognostic factors submitted to meta-analysis.

Prognostic factor	Studies	Pooled OR^a^	Lower limit 95% CI	Upper limit 95% CI	I^2^ (%)
Jaundice	5	8.27	4.99	13.71	12
HIV positive	11	4.60	3.24	6.54	27
Tuberculosis	5	4.06	1.83	9.01	62
Age >45 *vs*. age 15–45 years	9	3.69	2.72	5.02	53
Oedema	5	3.52	1.77	7.03	85
Bleeding	5	3.37	2.62	4.34	0
Low haemoglobin (≤6.5 g/dl)	8	3.26	2.16	4.93	83
Severe malnutrition (BMI <16 kg/m^2^ or Z score <-3 or WHZ score <-4)	10	2.42	2.07	2.85	0
Long duration of illness (≥2 months)	7	1.82	1.29	2.57	68
Age<5 *v*s. age 15–45 years	6	1.59	1.28	1.98	27
Large spleen size (≥10 cm)	8	1.27	1.02	1.56	30
Gender (female *vs*. male)	7	0.87	0.44	1.74	96
Relapse *v*s. primary VL	8	0.71	0.33	1.50	77
Age <15 *v*s. age 15–45	11	0.64	0.41	1.00	94

Abbreviations: BMI, body mass index; CI, confidence interval; HIV, human immunodeficiency virus; OR, odds ratio; VL, visceral leishmaniasis; WHZ, weight for height Z score.

^a^ OR from random effects model.

The individual forest plots, subgroup analyses, and funnel plots for each prognostic factor are given in the supplementary information (S2 Text). There were ten factors for which we found a statistically significant association with mortality: HIV, tuberculosis, young or old age, jaundice, oedema, bleeding, anaemia, severe malnutrition, long duration of illness, and pronounced splenomegaly. Jaundice was the strongest predictor of mortality, with a pooled OR of 8.27, followed by HIV infection (OR 4.60) and tuberculosis (OR 4.06) (Table 3). For factors such as age below five years, pronounced splenomegaly, and longer duration of illness, the association was less pronounced, with an OR below two (Table 3).

Heterogeneity across studies was high for most prognostic factors, with the exception of jaundice, HIV status, bleeding, malnutrition, age below five years, and spleen size (Table 3). However, despite the large heterogeneity, for all prognostic factors except relapse *vs*. primary VL, the associations were found to be in the same direction. For relapse *vs*. primary VL, the heterogeneity could be explained by the proportion of HIV-infected patients in the studies. Indeed, a subgroup analysis showed that in studies with >90% HIV-infected patients the odds of dying was more than three times lower in relapse than in primary VL (OR 0.29, 95% CI 0.13–0.61) while for the other studies with a lower HIV prevalence, there was no clear association (S2 Text). In subgroup analysis by country (S2 Text), for most variables there were no clear patterns between countries because of wide and overlapping confidence intervals. However, some associations were different for Sudan compared to the other countries. Children <15 years had lower odds of death in all countries except Sudan, while for Sudan the odds ratio of dying after long duration of illness was higher than in Ethiopia. In Sudan, females have lower odds of dying than males, in Ethiopia and South Sudan there was no significant difference, while in Uganda, females had a higher chance of death.

Six factors (diarrhoea, vomiting, weakness, malaria, tissue parasite load and treatment regimen) were studied in at least three but less than five studies (S2 Text). For diarrhoea and vomiting, all available studies showed an association with higher mortality, but these findings were not always significant. For weakness, malaria, and tissue parasite loads, the estimates went in opposite directions (S2 Text). Although many different studies incorporated treatment as a prognostic factor, a variety of regimens were compared in each study, allowing for only three comparisons. These showed that amphotericin deoxycholate is associated with higher mortality than antimonials, pentostam was associated with higher mortality compared to generic SSG (non-significant) and SSG alone with higher mortality than a combination of SSG and paromomycin (non-significant) (S2 Text).

There were only nine studies reporting multivariable analyses [17,23–26,38,60,71,72]. Table 4 gives an overview of the prognostic factors included in the models and indicates which of them remained significantly associated with death after adjusting for other factors.

**Table 4:** Overview of studies reporting multivariable analysis of factors associated with mortality in VL patients in East Africa.

Study^a^	HIV	Tuberculosis	Jaundice	Bleeding	Weakness	Anaemia	Malnutrition	Age >45 years	Age <5 years	Oedema	Ascites	Oedema or ascites	Spleen size	Duration of illness	Vomiting	Diarrhoea
Abongomera 2017 [71]	S	S	S	S	S	S	NS	NS	NS	S	S		NS	NS		
Lyons 2003 [25] HIV tested	S			S	NS		NS			NS			NS	NS	S	NS
Lyons 2003 [25] no HIV tested				S	S		S			NS			NS	NS	S	S
Herrero 2009 [24]	S	S				S		S	NS	S			NS		NS	
Mengistu 2007 [17]	S			S		NS	S	S								
Kamink 2017 [38] (≥19 years); HIV(-)			S		S	S	S	NS				S	NS	NS		
Kamink 2017 [38] (<19 years); HIV(-)			S		S	S	NS		S			NS	NS	NS		
Ritmeijer 2006 [60]	S														S	
Seaman 1996 [26]				NS		S	S	S	S				NS	S	NS	NS
Mueller 2009 [23]		S	S			NS	NS	S	S				S	NS		NS

Abbreviations: NS, not significant–factor included in multivariable analysis, was not significantly associated with mortality after adjustment for other factors; S, significant–factor included in multivariable analysis, remained significantly associated with mortality after adjustment for other factors; VL, visceral leishmaniasis.

^a^ A small study confined to HIV patients not included in the Table [72].

All multivariable analyses were based on logistic regression (S1 Table and S2 Table). Most predictors analysed were based on findings from previous studies. Predictor selection during modelling was described in only 4 [23,71,72,75] of the 9 studies reported in Table 4. Two studies developed and externally validated clinical prediction tools [38,71]. The procedures for validation and assessment of performance of the clinical prediction tools were fully described. As a measure of discrimination, areas under the receiver operating curve were reported: 0.83 for the Ethiopian study (adults only), 0.74 for the model including adults in Sudan and 0.83 for children/adolescents in Sudan. Calibration was not done.

The prognostic factors included in multivariable analyses differed widely across studies, precluding the conduct of a meta-analysis of adjusted measures of effect. Some factors were fairly consistently identified as prognostic factors in adjusted analyses, such as HIV, tuberculosis, jaundice, bleeding, weakness, and anaemia (Table 4). Other factors such as malnutrition, oedema/ascites, young/old age, vomiting, and diarrhoea were identified in some, but not in others. For factors such as spleen size and duration of illness, most studies did not find a significant association in adjusted analyses (Table 4).

There were only two studies that developed and validated a clinical prediction tool and reported absolute risks of mortality (Abongomera *et*. *al*. [71] and Kamink *et*. *al*. [38] in Table 4). One of these studies was conducted in Ethiopia in an area with a high HIV prevalence where VL is particularly common amongst young male HIV infected migrant workers [71]. The other study in South Sudan excluded HIV infected patients and developed a separate tool for children/adolescents and adults [38]. Even though a few factors such as bleeding and tuberculosis were not assessed in the South Sudanese study, independent prognostic factors were very similar in both studies, such as jaundice, anaemia, weakness, and oedema/ascites [38,71]. Factors such as spleen size and duration of symptoms were not retained in both studies. In a setting with high HIV prevalence in Ethiopia, HIV coinfection and tuberculosis were also retained as prognostic factors [71].

## Discussion

We conducted a systematic review and meta-analysis of prognostic factors for mortality in VL patients in East Africa. A total of 48 studies [17,21–26,33–73] were included, out of which 12 prognostic factors could be submitted to meta-analysis (sourced from 27 studies [17,21–26,33–47,60,65,71–73]). Out of these, ten factors were found to be significantly associated with mortality. HIV and tuberculosis are concurrent infections which are prevalent in East Africa. Other markers such as jaundice, oedema, pronounced splenomegaly, anaemia, bleeding, duration of illness, and malnutrition likely indicate severity and/or progression of the disease. Additionally, old and young age were also associated with mortality. Most of these prognostic factors can be easily identified by health professionals in resource-constrained settings. All of these were also identified as prognostic factors in a meta-analysis from Latin America [20], suggesting they apply across continents. Future prognostic studies within East Africa evaluating novel or other prognostic markers should consider these as “core” prognostic factors for inclusion in multivariable analysis.

While, ideally, adjusted estimates would have been calculated in meta-analysis, this was not possible due to the wide variability of prognostic factors included in multivariable analysis in the different studies. Only one study included all core factors [71]. Nevertheless, several of the core factors were found to retain their prognostic value in the studies performing multivariable analysis. The effect of age varied across the few studies [17,23–26,38,71].

We found two studies which developed clinical prediction tools [38,71]. The factors included in these two tools are very similar to those recently proposed in a clinical prediction tool in Brazil [20]. It remains to be defined whether the two tools also perform well in neighbouring countries within East Africa. We note that we could not include any studies on prognostic factors from Eritrea or Somalia.

Meanwhile, these tools could be used in routine practice for early detection of VL cases at high risk of death. This would require systematic assessment of several clinical factors and some laboratory testing (HIV, tuberculosis, haemoglobin). Patients with a predicted high risk of death could be triaged to the appropriate medical department or services providing more intensive care or closer patient observation. Those with a very low risk could be managed at the decentralised and/or ambulatory level. Nevertheless, impact studies are required to quantify the effectiveness of the implementation of such tools to reduce VL mortality.

Future studies could also aim to further improve the current prediction tools by adding a number of factors (especially laboratory tests) which were found predictive in other continents, but which are not yet (fully) assessed in East Africa. This includes renal impairment, elevated liver function tests, serum albumin levels, thrombocytopenia, and leukocytopenia [20]. However, some of these tests might not be available at the lower health care level in East-African countries. Diarrhoea and vomiting were retained as independent prognostic factors in the Latin American meta-analysis [20], but were not frequently studied in East Africa (and therefore not included in the present meta-analysis).

Bacterial coinfection was also found to be associated with mortality in several studies in Latin America [20]. No studies assessed this in East Africa. There was however one study which found sepsis to be associated with adverse outcomes, defined as death or treatment failure (composite outcome) [21]. Of interest, bacterial sepsis has been found in one in five patients in a study in Ethiopia [76]. Particularly given the increasing burden of multi-drug resistant bacterial infections globally, detailed studies on the prevalence, pattern, and prognostic significance of bacterial infections in VL in East Africa are merited.

Many of the studies used routine data and were retrospective in nature. Consequently, only a limited number of prognostic factors were assessed in most studies. Moreover, differences in definitions and cut-offs of prognostic factors or in the reliability of the clinical assessments likely exist across studies. This could explain the considerable heterogeneity found in the present review. However, despite this heterogeneity, the associations with mortality generally went in the same direction. Standardised prospective data collection within VL programs (or in selected sites) would improve the quality and consistency of the available data, and such an enhanced routine data collection system should include the core indicators of mortality. Furthermore, a recent initiative for data sharing for infectious diseases–including VL—could make the meta-analysis of individual patient data possible [77]. We did not have data on drug resistance. However this is probably of a lesser concern in the East African region, compared to the Indian subcontinent where high resistance rates have been reported [15].

### Conclusion

A total of 48 studies [17,21–26,33–73] were included with a total population of 150,072 VL cases from five countries. Ten prognostic factors were identified in the meta-analyses. These factors should be considered “core” prognostic factors in future prognostic studies. Several of these such as HIV, tuberculosis, jaundice, bleeding, and weakness remained significant after adjustment for other factors in the few studies conducting multivariable analysis [17,23–26,38,71]. One clinical prediction tool was developed in Ethiopia [71], another in South Sudan [38]. As both performed relatively well, future prognostic factor studies should evaluate whether these tools also have a good performance in neighbouring countries, and whether they can be further improved by adding additional prognostic factors not yet (fully) explored within East Africa.

## Supporting information

S1 Text Prisma checklist. (DOC)

S2 Text Forest plots, subgroup analyses, and funnel plots for each prognostic factor. *1. Forest plots displaying the crude odds ratios of the association between the prognostic factors and mortality for factors included in meta-analysis (at least five estimates); 2. Subgroup analysis according to HIV prevalence and Country; 3. Forest plots displaying the crude odds ratios of the association between the prognostic factors and mortality for factors with at least three but less than five estimates and mortality; 4. Funnel plots*.
(DOCX)

S1 Table Overview and summary of the observational studies included. (DOCX)

S2 Table Overview and summary of the trials included. (DOCX)

S1 Fig Assessment of risk of bias for 37 observational studies using the Quality in Prognostic Studies (QUIPS) tool. (*Figure structure*: *on top “studies included in the meta-analysis”–below*: *“studies included in narrative synthesis only”*).
(JPG)

S2 Fig Assessment of risk of bias for 11 trials using the Quality in Prognostic Studies (QUIPS) tool. (*Figure structure*: *on top “studies included in the meta-analysis”–below*: *“studies included in narrative synthesis only”*).
(JPG)
