# Supplementary material for: Prognostic factors for mortality among patients with visceral leishmaniasis in East Africa: Systematic review and meta-analysis
Source: PLoS Negl Trop Dis. 2020 May 15;14(5):e0008319. doi: 10.1371/journal.pntd.0008319 (PMC7255612; doi:10.1371/journal.pntd.0008319)
Supplement: S2 Text — 1. Forest plots displaying the crude odds ratios of the association between the prognostic factors and mortality for factors included in meta-analysis (at least five estimates); 2. Subgroup analysis according to HIV prevalence and Country; 3. Forest plots displaying the crude odds ratios of the association between the prognostic factors and mortality for factors with at least three but less than five estimates and mortality; 4. Funnel plots. (DOCX) [file pntd.0008319.s002.docx]

Prognostic factors for mortality among Patients with visceral leishmaniasis in East Africa: a meta-analysis

# Forest plots with meta-analysis

Figure 1: Forest plot: Age <5 vs 15-45

Figure 2: Forest plot: Age <15 vs 15-45

Figure 3: Forest plot: Age >45 vs 15-45

Figure 4: Forest plot: relapse VL versus primary VL

Figure 5: Forest plot: duration of illness

Figure 6: Forest plot: HIV status

Figure 7: Forest plot: Malnutrition

Figure 8: Forest plot: Haemoglobin

Figure 9: Forest plot: Spleen size

Figure 10: Forest plot: Gender

Figure 11: Forest plot: Oedema

Figure 12: Forest plot: Tuberculosis

Figure 13: Forest plot: Jaundice

Figure 14: Forest plot: Bleeding

# Forests plots of subgroup analyses

Figure 15: Forest plot: Age <15 vs 15-45

Figure 16: Forest plot: Age >45 vs 15-45

Figure 17: Forest plot: relapse VL versus primary VL

Figure 18: Forest plot: Age <5 vs 15-45

Figure 19: Forest plot: Age <15 vs 15-45

Figure 20: Forest plot: Age >45 vs 15-45

Figure 21: Forest plot: Duration of illness

Figure 22: Forest plot: gender

Figure 23: Forest plot: Haemoglobin

Figure 24: Forest plot: HIV

Figure 25: Forest plot: malnutrition

Figure 26: Forest plot: Oedema

Figure 27: Forest plot: relapse vs primary VL

Figure 28: Forest plot: spleen size

# Forest plots without meta-analysis

Figure 29: Forest plot: Weakness

Figure 30: Forest plot: Diarrhoea

Figure 31: Forest plot: Vomiting

Figure 32: Forest plot: Malaria

Figure 33: Forest plot: Parasite load

Figure 34: Forest plot: pentavalent antimonials (Sbv) vs amphotericin B deoxycholate (ABD)

Figure 35: Forest plot: Pentostam vs Sodium stibogluconate (SSG)

Figure 36: Forest plot: SSG vs SSG+paromomycin (PM)

# Funnel plots

Figure 37: Funnel plot: Age <5 vs 15-45

Figure 38: Funnel plot: Age <15 vs 15-45

Figure 39: Funnel plot: Age >45 vs 15-45

Figure 40: Funnel plot: Relapse VL vs primary VL

Figure 41: Funnel plot: duration of illness

Figure 42: Funnel plot: HIV status

Figure 43: Funnel plot: malnutrition

Figure 44: Funnel plot: Haemoglobin

Figure 45: Funnel plot: Spleen size

Figure 46: Funnel plot: Gender

Figure 47: Funnel plot: Oedema

Figure 48: Funnel plot: Tuberculosis

Figure 49: Funnel plot: Jaundice

Figure 50: Funnel plot: Bleeding
