# Supplementary material for: Prognostic factors for mortality among patients with visceral leishmaniasis in East Africa: Systematic review and meta-analysis
Source: PLoS Negl Trop Dis. 2020 May 15;14(5):e0008319. doi: 10.1371/journal.pntd.0008319 (PMC7255612; doi:10.1371/journal.pntd.0008319)
Supplement: S1 Table — (DOCX) [file pntd.0008319.s003.docx]

| **S1 Table. Overview and summary of the observational studies included** | | | | | | | | | |
| --- | --- | --- | --- | --- | --- | --- | --- | --- | --- |
| **Author (year)** | **Site (s)** | **Period of data collection** | **Study design** | **Inclusion /Exclusion criteria** | **Treatment given** | **Deaths/N** | **Univariate analysis: risk factors assessed** | **If multivariate analysis, risk factors included in final model** | **Multivariate analysis:**  **methods/**  **model development** |
| **In meta-analysis** | | | | | | | | | |
| Abongomera 2017 [71] | Abdurafi health centre; Ethiopia. | 1/2008 - 12/2013 | Retrospective cohort | Inclusion   - Outcome cure or in-health centre death   Exclusion   - Outcome transferred out, defaulter, or not reported | - AmB-based regimen (AmB alone or with MF for 28 days) - SSG-based regimen | 99/1,686 (5.9%) | - Age in years (<5, 5-18, >18-40, >40) - HIV status - Duration of illness (<2 or >2 months) - Severe malnutrition [BMI<16.0 kg/m^2^ (>19 years); BMI for age Z score<–3 (5–19 years); weight for length/ height Z score<–3 (<5 years)] (NS) - Haemoglobin (<6.5 or >6.5g/dl) - Spleen size (<11 or >11 cm) - Relapse/primary VL - Oedema - Weakness (severe/collapse) - Bleeding - Jaundice - Tuberculosis - Ascites | - Age^a^ - HIV status - Haemoglobin - Oedema - Bleeding - Jaundice - Tuberculosis - Ascites | - Spiegelhalter method: logistic regression with factors - selected based on likelihood ratios - (<0.5/>2 in univariate; <0.67/>1.5) in multivariate analysis (backward elimination) - Prognostic prediction model developed - AUROC (discrimination): 0.83 - No calibration - External validation: AUROC 0.78 |
| Abongomera 2018 [72] | MSF-supported health centre in Abdurafi; Ethiopia. | 1/2011 - 8/2014 | Retrospective cohort | Inclusion   - HIV-VL coinfection - Adults - Treatments with AmB infusion and oral MF for 28 days   Exclusion   - Treatment discontinuation - Defaulters - Patients with missing outcomes - Patients who transferred out | AmB 30 mg/kg total dose with MF 100 mg/day for 28 days | 22/173 (12.7%) | - Age in years (18-40 vs >40)* - Duration of illness (< or > 2 months) (NS) - BMI (< or >16kg/m^2^) (NS) - Haemoglobin (< or >6.5g/dL)* - Spleen size (< or >11cm) (NS) - Primary/Relapse * - Oedema and/or ascites (NS) - Weakness (severe/collapse) (NS) - Bleeding (NS) - Jaundice (NS) - Tuberculosis (NS) - ART use (NS) - Parasite grade (< or =6+)* - Advanced HIV (WHO stage IV or CD4<50) (NS) | - Age* - Haemoglobin* - Primary/Relapse VL* | - Logistic regression with factors with P <0.1 in univariate included, back-ward elimination (P<0.05) - No prediction model developed |
| Adam 2016 [73] | Unspecified sites in Gadarif State; Sudan. | 1/2002 - 12/2015 | Retrospective cohort | Inclusion   - VL cases from ministry of health of Gadarif State database | - First line: 20 mg/kg SSG with 15 mg/kg paromomycin for 17days - Second line: AmB 3mg/kg for 10 days | 1,686^b^/51,773^c^ (3.3%) | - Age in years (NS) - Sex (NS) - Education (> or < secondary) (NS) - Rural residence* |  |  |
| Adam 2017 [33] | Gadarif hospital; Sudan. | 1/2014 - 12/2015 | Prospective cohort | Inclusion   - Pregnant women with VL - Provided written informed consent | Not reported | 8/45 (17.8%) | - Age in years (NS) - Duration of illness (< or >1 month)* - Parity (NS) - Pregnancy duration (NS) - Rural residence* - Illiteracy (NS) - Black race* - Attending prenatal care (NS) | - Age (NS) - Duration of illness* - Parity (NS) - Pregnancy duration (NS) - Rural residence* - Illiteracy (NS) - Black race* - Attending prenatal care (NS) |  |
| Aderie 2017 [34] | Abdurafi health centre; Ethiopia. | 2008 - 2015 | Retrospective cohort | Inclusion   - VL patients with HIV infection - Started on ART at Abdurafi health centre from 2008-2015 | - SSG for 30 days - AmB 6-10 doses - AmB 6-10 doses with MF for 28 days - MF for 28 days | 20/213 (9.4%) | - Relapse/primary VL - Time of ART initiation (before/after VL episode)* |  |  |
| Atia 2015 [35] | Tabarak Allah hospital; Sudan. | 7/2011 - 1/2014 | Retrospective cohort | Inclusion   - Lab confirmed VL - Age 2-45 years   Exclusion   - HIV infection - Pregnancy - SSG contraindications | SSG 20 mg/kg with PM 15 mg/kg for 17days | 6/809 (0.7%) | - Relapse/primary VL |  |  |
| Collin 2004^d^ [22] | MSF treatment centres in Wudier, Lankien, Magang, Nimne, Thonyor; South Sudan. | 10/1998 - 5/2002 | Retrospective cohort | Inclusion   - All VL patients with treatment cards archived at the MSF administrative centre in Kenya   SSG contraindications | - SSG 20 mg/kg - SSG 20 mg/kg with PM 15 mg/kg - SSG with AmB - SSG with PM and AmB | 255^e^/3,365 (7.6%) | Patients >=16 years old   - Age in years (16-24, 25-34, 35-44, >45) - Duration of illness (<2 , 2-4 , >5 months)* - Haemoglobin (<8, 8.0-10.9, >11 g/dl)* - BMI (<13, 13.0-14.0, 15.0-16.9, >17 kg/m2)*   Patients <16 years old   - Age in years (<2, 2-4, 5-9, 10-15)* - Haemoglobin (<6, >6 g/dl)* - Spleen size (Hackett grade 0, 1-2, 3-5)* - Weight for height (<60%, 60.0-69.9%, 70.0-79.9%, >80%)   Overall^f^   - Bleeding* - Diarrhoea* - Vomiting* | Unclear, it is likely multivariate analysis was not done, but only analysis adjusted by sex |  |
| Diro 2014 [36] | LRTC Gondar; Ethiopia. | 11/2011 - 1/2013 | Retrospective cohort | Inclusion   - HIV infection - Treated with antimonials | SSG 20 mg/kg for 30 days | 8/57^c^ (14.0%) | - BMI (< or >18.5 kg/m^2^) (NS) - Spleen size (< or >10 cm) (NS) - Relapse/primary VL (NS) - ART use (NS) - Baseline CD4 count (< or >100 cell/µL) (NS) - Parasite density (<4 or >4) (NS) |  |  |
| Hailu 2010 [37] | Gondar University hospital; Arba Minch hospital; Ethiopia. | 1/2008 - 2/2009 | Retrospective cohort | Inclusion   - VL treated at the inclusion centres | Glucantime 20 mg/kg for 30 days | 3/52^g^ (5.8%) | - HIV status (positive, negative, unknown) |  |  |
| Herrero 2009^b^ [24] | Addis Zemen health centre; Ethiopia. | 25/5/2005 - 13/12/2007 | Retrospective cohort | Inclusion   - VL diagnosis and treatment at Addis Zemen health centre   Exclusion   - Relapse VL - Outcome data missing or ambiguous | - First line: SSG - Second line: AmB alone or with SSG | 87/2,177 (4.0%) | - Age in years (<5, 5-14, 15-29, 30-44, 45-80)* - Sex (NS) - HIV status (positive, negative, unknown)* - Malnutrition [BMI <16 kg/m^2^ (age >19), BMI Z score <-3 (age 5-19), oedema or Weight for height Z score <-3 (age <5)]* - Haemoglobin (< or >7 g/dl)* - Spleen size (below median for age, 50-75th percentile, >75th percentile) (NS) - Vomiting* - Oedema* - Tuberculosis* - Pneumonia* | - Age - Oedema* - Haemoglobin* - HIV status* - Tuberculosis* |  |
| Hurissa 2010 [21] | Gondar University hospital; Kasay Abera hospital; Ethiopia. | 1/2006 - 12/2008 | Retrospective cohort | Inclusion   - Age > 15 years - Lab confirmed VL | - First line: MA or SSG 20 mg/kg for 28-30 days - Second line: AmB 3 mg/kg for 6-10 days | 24/241 (10.0%) | - HIV status |  |  |
| Kamink 2017 [38] | MSF hospital in Lankien; South Sudan. | 7/2013 - 6/2015 | Retrospective cohort | Inclusion   - VL patients   Exclusion   - Incomplete data - Defaulters - HIV infection | - First line: SSG 20 mg/kg with PM 15 mg/kg for 17 days - Second line: AmB 5 mg/kg for 6 alternate days | 186/6633 (2.8%) | Children and adolescents (<19 years)   - Age in years (<2, 2-5, 6-18)* - Sex (NS) - Duration of illness (<1, 1, >2 months) (NS - Malnutrition (Weight for height Z score <-4, =-3, >-2)* - Haemoglobin (<6, 6-7.4, 7.5-8.9, >9 g/dl)* - Spleen size (<1, 1-4, 5-7, >7 cm) (NS) - Relapse/primary VL (NS) - Oedema/ascites* - Weakness (normal, severe, state of collapse)* - Jaundice* - Lymphadenopathy*   Adults (>=19 years)   - Age in years (18-25, 26-35, 36-45, >45)* - Sex (NS) - Duration of illness (<1 month, 1 month, >2 months) (NS) - BMI (<13, 13-14.4, 14.5-15.9, >16 kg/m^2^)* - Haemoglobin (<6, 6-7.4, 7.5-8.9, >9 g/dl)* - Spleen size (<1, 1-4, 5-7, >7 cm)* - Relapse/primary VL (NS) - Oedema/ascites* - Weakness (normal/severe/state of collapse)* - Jaundice* - Lymphadenopathy (NS) | Children and adolescents (<19 years)   - Age* - Weakness* - Haemoglobin* - Jaundice*   Adults (>=19 years)   - Weakness* - BMI* - Haemoglobin* - Oedema/ascites* - Jaundice* | Logistic regression  Variables with P<0.2 in univariate included in multivariate analysis  Backward elimination  Prognostic prediction model developed   - AUROC (discrimination): adults: 0.74; children: 0.83 - No calibration   External validation: adults AUROC 72.2%, 79.5%, 71.2%; children AUROC 72.2%, 82.8%, 76.6 |
| Khalil 1998 [39] | El Gerief University hospital; Soba University hospital; Sudan. | 1/1989 - 12/1995 | Retrospective cohort | Inclusion   - Partial or no response to SSG | - Allopurinol 5 mg/kg with SSG 20 mg/kg for 30 days - Pentamidine 4 mg/kg 3x/week for 40 days - Ketoconazole 400m/day with allopurinol 4-6 weeks - AmB 3 mg/kg for 14 days - Itraconazole 200 mg/day with allopurinol 5 mg/kg/day for 6 weeks - Pentamidine 15 mg/kg for 20 days | 9/33 (27.3%) | - Tuberculosis |  |  |
| Kimutai 2017 [40] | Multi-country study: Sudan (2), Ethiopia (2), Kenya (1), Uganda (1). | 4/2011 - 11/2013 | Prospective cohort | Inclusion   - Treatment with SSG/PM combination | SSG 20 mg/kg with PM 15 mg/kg for 17 days | 32/3,126 (1.0%) | - Age (NS) |  |  |
| Lyons 2003 [25] | Unspecified MSF VL clinic in Tigray, Ethiopia. | 12/1998 - 5/2000 | Retrospective cohort | Inclusion   - Primary VL - Outcome cure or death   Exclusion   - PKDL - Defaulters - Transfer out | SSG 20 mg/kg for 30 days | 146/791 (18.5%) | - Age in years (0-14, 15-45, >45)* - Sex (NS) - HIV* - Duration of illness (0-8, 9-16, 17-24, 25-48 weeks) - BMI (< or >16 kg/m^2^)* - Diarrhoea* - Vomiting* - Oedema - Weakness* - Bleeding* - Jaundice* - Neurological complication* | - Sex (NS) - HIV* - Duration of illness (NS) - BMI* - Diarrhoea* - Vomiting* - Oedema (NS) - Weakness* - Bleeding* - Spleen size (NS) - Liver size (NS) | Logistic regression  Method for selection of predictors for inclusion in analysis unclear, method for selection process in multivariate analysis unclear (presumably backward elimination)   - No prediction model developed |
| Maru 1979 [41] | Public health college of Gondar hospital; Ethiopia. | 5/1971 - 2/1972 | Cohort (not clear whether prospective or retrospective) | Inclusion   - Lab confirmed VL | - Pentostam 400 mg/day (up to 6 g total dose) - Neostibosam (Ethylstibamine) 2.7 g | 3/18 (16.7%) | - Malnutrition - Diarrhoea |  |  |
| Mengesha 1978 [42] | Gondar hospital; Ethiopia. | 9/1973 - 8/1974 | Prospective cohort | Inclusion   - Lab confirmed VL | - Pentostam 10 mg/kg for 30 days - Glucantime for 14 days | 15/27 (55.6%) | - Age in years - Sex - Haematocrit - White blood cells - % Lymphocytes - Platelets - Albuminuria - Serum protein in g albumin/globulin - Formol gel test |  |  |
| Mengistu 2007 [17] | Gondar university hospital; Ethiopia. | 1/1999 - 7/2004 | Prospective cohort | Inclusion   - All VL patients | SSG for 30 days | 52/221 (23.5%) | - Age in years (< or >20)* - HIV status* - BMI (<15, >15 kg/m^2^)* - Bleeding* - Haematocrit (<15%, >15%)* - Dark/grey skin discolouration* | - Age* - HIV status* - BMI* - Bleeding* - Haematocrit - Dark/grey skin discolouration |  |
| Mohammed 2016 [43] | Gedarif teaching hospital; Sudan. | 1/2013 - 6/2014 | Retrospective cohort | Inclusion   - All VL patients | Not reported | 27^b^/313 (8.6%) | - HIV status - Hepatitis B - Hepatitis C Malaria - HIV/Hepatitis B/Hepatitis C/Malaria coinfection |  |  |
| Mueller 2009 [23] | Amudat hospital; Uganda. | 1/2000 - 12/2005 | Retrospective cohort | Inclusion   - Lab confirmed VL - Primary VL | - First line: MA 20 mg/kg for 30 days, later replaced by pentostam 20 mg/kg 30 days - Second line: ampB deoxycholate 1 mg/kg alternate days for 30 days | 68/1858 (3.7%) | - Age in years (0-5, 6-15, 16-45, >45)* - Sex* - Duration of illness (0-5, 6-10, >10 weeks) - BMI (<13, 13-14, 14.1-15, 15.1-16, 16.1-17, 17.1-18, >18 kg/m2) (NS) - Haemoglobin (<5.3, 5.3-6.7, 7.3-10.7, >11 g/dl) (NS) - Spleen size (<11, 11- 14 or >14 cm) - Diarrhoea* - Tuberculosis* - Acute respiratory infection (NS) - Ear, nose, throat infection (NS) - Distance to hospital (0-15, 16-50, 51-100, >100 km) - Weight over height (<70, 70-79, 80-89, >90 %) (NS) - Liver disease* - Adverse events* - Pregnancy* - Malaria (NS) | - Age* - Spleen size (< or >14 cm) - Haemoglobin - Liver disease* - Tuberculosis* - Pregnancy (NS) - Adverse events* | Logistic regression  Inclusion of variables with P<0.2 in univariate analysis  (P-value not mentioned)  No prediction model developed |
| Omar 2016 [44] | Gadarif hospital; Sudan. | 1/2015 - 12/2015 | Retrospective cohort | Inclusion   - Notified cases of VL reported - Known HIV status | AmB 3 mg/kg 10 days | 3^b^/659 (0.5%) | - HIV status* |  |  |
| Ritmeijer 2011 [45] | MSF clinics in Humera and Abdurafi; Ethiopia. | 1/2007 - 1/2009 | Retrospective cohort | Inclusion   - Severely ill or HIV(+) VL - AmB monotherapy   Exclusion   - Patients who switched to AmB after SSG toxicity - Unknown HIV status | AmB total 30 mg/kg in 6 doses on alternate days | 19/289 (6.6%) | - HIV status (NS) - Relapse/primary VL (NS) |  |  |
| Salih 2014 [46] | Tabarak Allah hospital; Sudan. | 3/2010 - 12/2012 | Retrospective cohort | Inclusion   - Lab confirmed VL - Treatment with AmB | AmB total 30 mg/kg in 10 doses of 3 mg/kg on consecutive days | 15/379 (4.0%) | - Relapse/primary VL |  |  |
| Seaman 1996 [26] | MSF VL treatment centre in Duar; South Sudan. | 8/1990 - 7/1991 | Retrospective cohort | Inclusion   - Primary VL - No previous VL treatment - Medical records found | SSG 20 mg/kg for 30 days | 336/3076 (10.9%) | - Age in years (<5, 5-14, 15-24, 25-34, 35-44, >45; <18, >18)* - Sex (NS) - Duration of illness (< or >5 months)* - BMI (<12, 12.0-12.9, 13.0-13.9, 14.0-14.9, 15.0-15.9, 16.0-16.9, 17.0-17.9, >18 kg/m^2^) (NS) - Haemoglobin (<60, 60-69, 70-79, 80-89, 90-99, >100 g/l)* - Spleen size (Hackett grade 1, 2, 3, 4-5) (NS) - Diarrhoea (NS) - Vomiting* - Bleeding (NS) - Parasite density grade* | - Age* - Sex (NS) - Duration of illness* - Haemoglobin* - BMI (NS) | Logistic regression  Multivariate analysis unclear  No prediction model developed |
| Zijlstra 1992  [47] | Kala azar hospital, Khartoum; Sudan. | 1/1989 - 2/1990 | Retrospective cohort | Inclusion   - All VL cases | - SSG 10 mg/kg for 30 days - SSG 20 mg/kg for 15 days | 78^b^/693 (11.3%) | - Children (< 15 years) vs adults (> 15 years) |  |  |
| **Not in meta-analysis** | | | | | | | | | |
| Boateng 2017 [48] | Unspecified VL reporting sites from South Sudan, one from the South, one from North. | 2009 - 2013 | Retrospective cohort | Inclusion   - VL patients registered on line lists from treatment sites   Exclusion   - Case records without variables of interest | - AmB - MF - SSG - SSG with PM - AmB with MF | 91^b^/2,466^c, h^ (3.7%) | - Age* - Duration of illness* - Relapse VL/primary VL/PKDL* |  |  |
| Chulay 1985 [49] | Unspecified site in Kenya. | 10/1980 - 3/1983 | Prospective cohort | Not described | SSG as follows   - 10 mg/day for 30 days - 20 mg/day for 15 days - 30 mg/day for 10 days - 30 mg/day for 15 days - 30 mg/day for 30 days - 20 mg/day for 60 days - 40 mg/day for 30 days - 40 mg/day for 60 days - 60 mg/day for 30 days | 3/59^i^ (5.1%) |  |  |  |
| Gorski 2010 [50] | MSF treatment centres in Upper Nile State, Jonglei State, and Unity State; South Sudan | 1999 - 2007 | Retrospective cohort | Inclusion   - Patients with data entered in the VL treatment database | - SSG 20 mg/kg with PM 15 mg/kg for 17 days - SSG 20 mg/kg for 30 days | 34/621^j^ (5.5%) |  | - VL treatment* - Age, sex, year and treatment centre were added as potential confounders | Logistic regression  Variables with P <0.05 in univariate & a-priori defined variables included in multivariate analysis;  Full model  No prediction model developed |
| Kager 1984 [51] | Kenyatta national hospital; Kenya. | 8/1978 - 3/1980 | Prospective cohort | Inclusion   - All VL patients | - Pentostam 10 mg/kg for 30 days - Allopurinol 15-30 mg/kg divided in 3 doses with Pentostam 10 mg/kg in different durations | 2/64 (3.1%) |  |  |  |
| Melaku 2007 [52] | MSF treatment centres in Eastern Upper Nile region & Western Upper Nile region; South Sudan. | 2002 - 2005 | Retrospective cohort | Inclusion   - Treatment with 30 days of SSG or 17 days of SSG & PM   Exclusion   - Relapse VL - Defaulters | - SSG at 20 mg/kg for 30 days - SSG at above dosage with PM at 15 mg/kg for 17 days | 182/4.263 (4.3%) | -Sex (NS) | Treatment regimen, stratified by age < or >5 years, adjusted for malnutrition, treatment centre, walking status, length of illness | Logistic regression  Multivariate analysis unclear  No prediction model developed |
| Mueller 2008 [53] | Amudat hospital; Uganda. | 9/2003 - 4/2004 (AmB cohort)  9/2002 - 4/2003 (Historical MA cohort ) | Retrospective cohort comparing AmB cohort with historical cohort treated with MA | Inclusion   - Patients treated with AmB or MA) - Primary VL (only for historical cohort) | - AmB 1 mg/kg on alternate days for 30 days^k^ - MA 20 mg/kg for 30 days | 16/371 (4.3%) |  |  |  |
| Mueller 2014 [54] | Amudat hospital; Uganda; VL treatment centre in Kacheliba; Kenya. | Amudat: 2000 - 2006^l^  Kacheliba: 2006 - 2010 | Retrospective cohort | Inclusion   - Primary VL | - AmB 1mg/kg on alternate days for 15 days - MA or SSG, 20 mg/kg for 30 days | 96^b^/4,605 (2.8%) |  |  |  |
| Ritmeijer 2003 [55] | Emergency hospital in Khartoum; Ler Hospital; VL treatment centres in Duar, Malakal, Um el Kher; Sudan. | 1989 - 2003 | Retrospective cohort | Not described | - SSG 20 mg/kg for 30 days - SSG 20 mg/kg with PM 15 mg/kg for 17 days | 3,900/>51,000^m^ (7.6%) | - Age* - Duration of illness* - Malnutrition* - Haemoglobin* - Vomiting* - Bleeding* |  |  |
| Tamiru 2016  [56] | LRTC, Gondar; Ethiopia. | 1/2009 - 12/2014 | Retrospective cohort | Inclusion   - VL patients treated with AmB   Exclusion   - Incomplete data - HIV infection | - Low dose AmB (<24 mg/kg total) - High dose AmB (24-35 mg/kg total dose) | 7/147 (4.8%) |  |  |  |
| Van Griensven 2017 [57] | Metema district hospital; LRTC, Gondar; Ethiopia. | Metema: 2010 - 2014  Gondar: 2010 - 2015 | Retrospective cohort | Inclusion   - VL-HIV coinfected patients   Exclusion   - No information on timing of HIV or VL diagnosis | - Antimonials - AmB 30 mg/kg with or without MF | 30/170 (17.6%) | - VL after HIV diagnosis vs. concurrent HIV/VL diagnosis |  |  |
| Van den Bogaart 2012 [58] | Amudat hospital; Uganda. | 1/2000 - 12/2006 | Case control | Inclusion:   - Cases: lab confirmed VL and malaria at or during hospital admission - Controls: lab confirmed VL with negative smear for malaria | - MA - AmpB deoxycholate - Pentostam | 72/2,414 (3.0%) | - Malaria infection (NS) |  |  |
| Van den Bogaart 2013 [59] | Primary dataset: Al-azaza kala-azar clinic, Gedarif teaching hospital; Tabarakallah hospital; Sudan.  Secondary dataset:  MSF treatment sites at Um-el-Kher and Kassab hospitals; Sudan. | Primary dataset: 1/2005 - 12/2010  Secondary dataset:  11/1998 - 12/1998 | Case control on two separate datasets | Inclusion:  Primary dataset:   - Cases: lab confirmed VL and malaria at hospital admission or during hospitalization - Controls: lab confirmed VL with negative smear for malaria - Secondary dataset: primary VL only | - Pentostam 20 mg/kg for 30 days - SSG 20 mg/kg for 30 days | Primary dataset 41/1,295 (3.2%)  Secondary dataset  22/516 (4.3%) | Primary dataset   - Malaria infection (NS)   Secondary dataset   - Malaria infection* |  |  |

Abbreviations: AmB, AmBisome; ART, anti-retroviral therapy; AUROC: Area under the receiver operator curve; BMI, body mass index; HIV, human immunodeficiency virus; IM, intramuscular; IV, intravenous; LRTC, leishmania research and treatment centre; MA, meglumine antimoniate; MF, miltefosine; MSF, Médecins Sans Frontières; PKDL, post kala-azar dermal leishmaniasis; PM, paromomycin; SSG, sodium stibogluconate; VL, visceral leishmaniasis.

**For univariate and multivariate analysis, * indicates significance, NS was not significant, nothing is indicated after a factor when significance was or could not be assessed**

^a^Although this paper did not assess for significance for the univariate and multivariate risk factor analysis, all variables included in the multivariate analysis were independant factors in the clinical score

^b^Not completely clear if this study reports only in-hospital deaths

^c^Includes patients who were lost (defaulted, referred or missing data)

^d^It is not clear whether patients were treated inpatient or outpatient

^e^Total deaths are not consistent in paper, ranging from 249-255. Denominator also varies, from 3,261-3,365

^f^Not clear whether this also includes PKDL patients

^g^Total number of patients analysed is unclear from text, ranging from 50-52

^h^Also includes several PKDL patients

^i^Includes 3 CL patients

^j^This refers only to the relapse population, and it is not completely clear whether 34 deaths occurred among all 621 relapse patients or just among the 166 relapse patients who had a record of their primary VL Treatment

^k^This comparison uses two different groups: the AmBisome group contains both relapse and primary VL patients, while the MA group only refers to primary VL patients

^l^There is overlap with the other papers written by Mueller, only the data from 2006 from Amudat and the data from Kacheliba are new

^m^3,900 refers to deaths in primary VL patients, while >51,000 refers to both primary VL, relapse VL and PKDL.
