# Supplementary material for: Prognostic factors for mortality among patients with visceral leishmaniasis in East Africa: Systematic review and meta-analysis
Source: PLoS Negl Trop Dis. 2020 May 15;14(5):e0008319. doi: 10.1371/journal.pntd.0008319 (PMC7255612; doi:10.1371/journal.pntd.0008319)
Supplement: S2 Table — (DOCX) [file pntd.0008319.s004.docx]

| **S2 Table. Overview and summary of the trials included.** | | | | | | | | | |
| --- | --- | --- | --- | --- | --- | --- | --- | --- | --- |
| **Author (year)** | **Site (s)** | **Period of data collection** | **Study design** | **Inclusion /Exclusion criteria** | **Treatment given** | **Deaths/N** | **Univariate analysis: risk factors assessed** | **If multivariate analysis, factors included in final model** | **Multivariate analysis:**  **methods/**  **model development** |
| **In meta-analysis** | | | | | | | | | |
| Ritmeijer 2001 [65] | Temporary treatment centre in Densha; Ethiopia. | 11/1998 - 4/1999 | Randomised clinical trial | Inclusion   - Clinical and lab confirmed VL   Primary VL | - Pentostam 20 mg/kg for 30 days - SSG (generic 20 mg/kg for 30 days(NS) | 48/199 (24.1%) | - HIV status* |  |  |
| Ritmeijer 2006 [60] | Humera hospital; Mycadra health centre; Ethiopia. | Not available | Randomised clinical trial | Inclusion   - Males aged >15 years - Lab confirmed VL   Exclusion   - Severe comorbidity (likely to die during the month’s treatment) | - MF 100 mg for 28 days - SSG 20 mg/kg for 30 days* | 36/580 (6.2%) | - Age* - HIV (positive, unknown, negative)* - Vomiting* - BMI* - Haemoglobin* - Diarrhoea* - Inability to walk unaided* | - Age - HIV* - Vomiting* - BMI - Haemoglobin - Diarrhoea - Inability to walk unaided - VL treatment regimen* - MF - SSG | - Multivariate logistic regression - Predictors analysed were based on literature from previous studies. - Predictor selection during modelling were presumably by backward elimination. - No prediction model developed. |
| **Not in meta-analysis** | | | | | | | | | |
| Hailu 2010 [61] | Multi-country study: Sudan (2), Ethiopia (2), Kenya (1). | 11/2004 - 1/2010 | Randomised clinical trial | Inclusion   - Aged 4-60 years - Lab confirmed VL   Exclusion   - Antileishmanial drug <6 months - Severe protein or caloric malnutrition - Previous hypersensitivity to SSG or aminoglycosides - Other concurrent conditions (see paper for details) | - PM 15 mg/kg for 21 days - SSG or 20 mg/kg for 30 days | 2/270 (0.7%) |  |  |  |
| Khalil 1998 [62] | Soba University hospital; Sudan. | Not available | Non-randomised clinical trial (started as randomised trial and then changed to non-randomised) | Inclusion   - Lab confirmed VL - First episode of VL   Exclusion   - Serious concomitant disease (HIV, TB, renal or cardiac disease) - Relevant outliers of safety and laboratory parameters - Pregnant or lactating women | - AmB 3 mg/kg for 14 days - SSG 20 mg/kg for 28 days | 1/26 (3.8%) |  |  |  |
| Moore 2001 [63] | Ortum mission hospital; Kenya. | 6/1996 - 7/1998 | Non-randomised clinical trial | Inclusion   - Primary VL - Lab confirmed VL   Exclusion   - Previous use of antimonials | - Pentostam 20 mg/kg for 30 days - SSG (generic) 20 mg/kg for 30 days (NS) | 4^a^/102 (3.9%) |  |  |  |
| Musa 2012 [64] | Multi-country study: Sudan (2), Ethiopia (2), Kenya (1), Uganda (1). | 11/2004 - 1/2010 | Randomised clinical trial | Inclusion   - 4-60 years old - Lab confirmed VL   Exclusion   - Taken antileishmanial drug <6 months - Severe protein or caloric malnutrition - Previous hypersensitivity reaction to SSG or aminoglycosides - Other concurrent conditions (except HIV) (see paper for details) | SSG *vs* PM   - SSG 20 mg/kg for 30 days - PM 20 mg/kg for 21 days   SSG *vs* SSG & PM   - SSG 20 mg/kg for 30 days - SSG 20 mg/kg and PM 15 mg/kg for 17 days | SSG vs PM  2/410 (0.5%)  SSG vs SSG & PM  4/767 (0.5%) |  |  |  |
| Seaman 1993 [66] | Treatment centre Duar; Sudan. | 12/1991 - 2/1992 | Randomised clinical trial | Inclusion   - Suspected VL with no prior treatment - Fever for >1 month - Palpable splenomegaly - Positive direct agglutination test >1:25,600 | - SSG 20 mg/kg for 30 days - PM 15 mg/kg and SSG 20 mg/kg for 17 days | 11/200 (5.5%) |  |  |  |
| Seaman 1995 [67] | Three treatment centres for VL, Western Upper Nile; South-Sudan. | 2/1993 - 1/1994 | Non-randomised clinical trial | Inclusion  Patients for regimens 1 & 2   - Relapse after treatment with combined Sbv and aminosidine - Incomplete parasitological response to Sbv and aminosidine - Severe illness   Patients for regimen 3   - Primary VL - No previous antileishmanial treatment | AmB:   - Regimen 1: 3 doses of 3-5 mg/kg given on days 0, 3, 10 - Regimen 2: Total 35 mg/kg on days 0, 3, 6, 8, 10 and 13 - Regimen 3: 4-5 mg/kg on days 0, 5, and 7 | 6/49 (12.2%) |  |  |  |
| Veeken 2000 [68] | MSF kala-azar treatment centres Um Kurraa and Kassab; Sudan. | 11/1998 - 12/1998 | Randomised clinical trial | Inclusion   - Primary VL   Exclusion   - Nomadic patients | - Pentostam 20 mg/kg for 30 days - SSG (generic) 20 mg/kg for 30 days(NS) | 22/516 (4.3%) |  |  |  |
| Wasunna 2016 [69] | Kimalel health centre; Kenya. Dooka and Kassab hospitals; Sudan. | 5/2010 - 2/2012 | Randomised clinical trial | Inclusion   - HIV negative - Aged 7-60 years - Lab confirmed VL - Primary VL - Signed informed consent   Exclusion   - Received anti-leishmanial drugs <6 months - Severe protein and caloric malnutrition - Females of child-bearing age - Other concurrent conditions (see paper for details) | - AmB 10 mg single dose with SSG 20 mg/kg for 10 days - AmB 10 mg single dose with MF 2.5 mg/kg for 10 days - MF 2.5 mg/kg for 28 days | 1/151 (0.7%) |  |  |  |
| Zijlstra 1993 [70] | Kala-azar hospital Khartoum; Sudan. | 9/1989 - 09/1990 | Randomised clinical trial | Inclusion   - Lab confirmed VL - Primary VL - Signed informed consent   Exclusion   - Other major diseases, such as renal or haematological disease | - SSG 10 mg/kg for 30 days - SSG 20 mg/kg for 30 days - SSG 20 mg/kg for 15 days | 7/104 (6.7%) |  |  |  |

Abbreviations: BMI, body mass index; HIV, human immunodeficiency virus; LRTC, leishmania research and treatment centre; MF, miltefosine; MSF, Médecins Sans Frontières; PM, paromomycin; Sbv, pentavalent antimonials; SSG, sodium stibogluconate; VL, visceral leishmaniasis.

**For univariate and multivariate analysis, * indicates significance, NS was not significant, nothing is indicated when significance was or could not be assessed**

^a^Not completely clear if this study reports only in-hospital deaths
