# Supplementary figures and images for: Prognostic factors for mortality among patients with visceral leishmaniasis in East Africa: Systematic review and meta-analysis
Source: PLoS Negl Trop Dis. 2020 May 15;14(5):e0008319. doi: 10.1371/journal.pntd.0008319 (PMC7255612; doi:10.1371/journal.pntd.0008319)

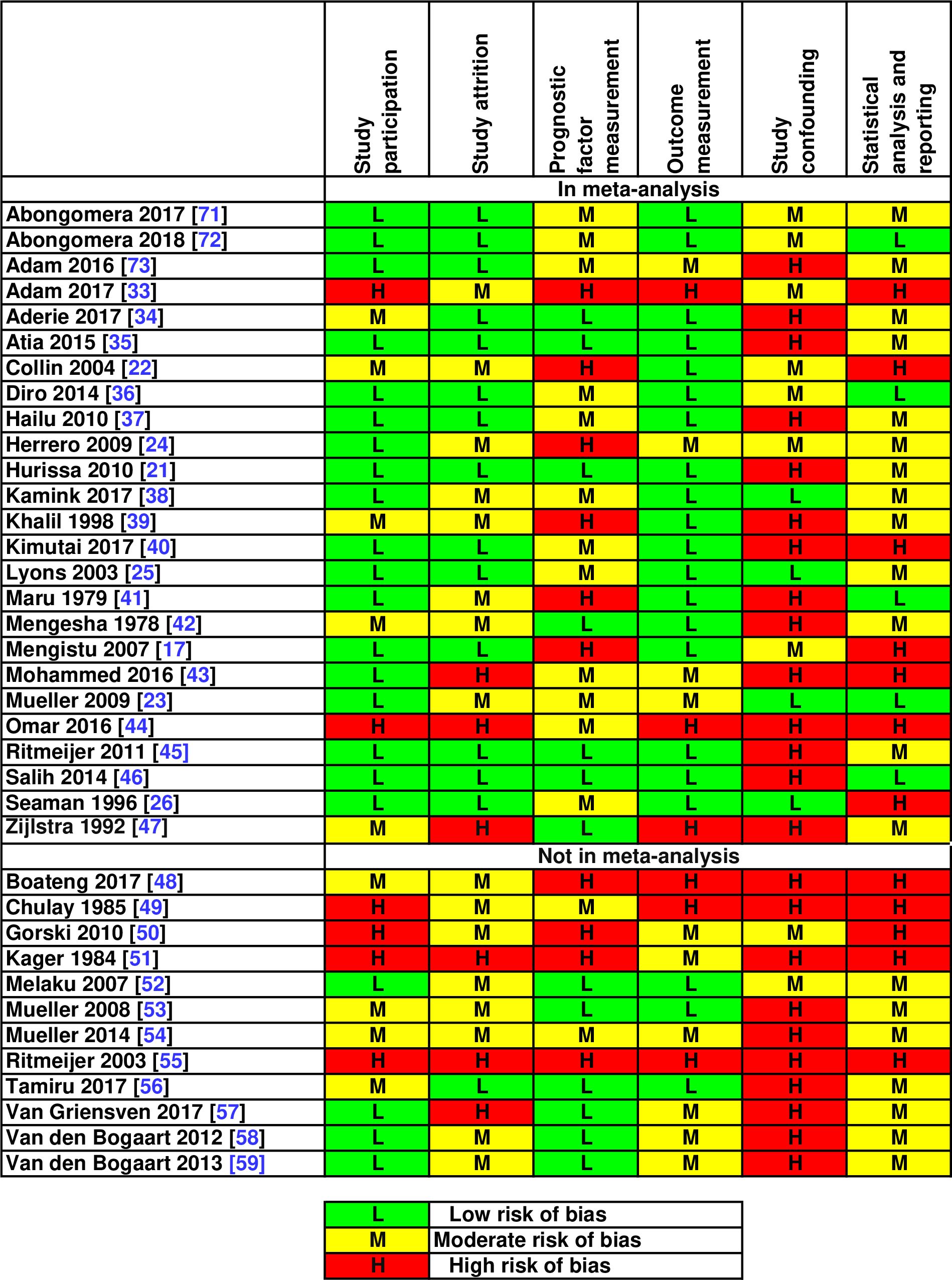

Supplement: S1 Fig — (Figure structure: on top “studies included in the meta-analysis”–below: “studies included in narrative synthesis only”). (JPG) [file pntd.0008319.s005.jpg]

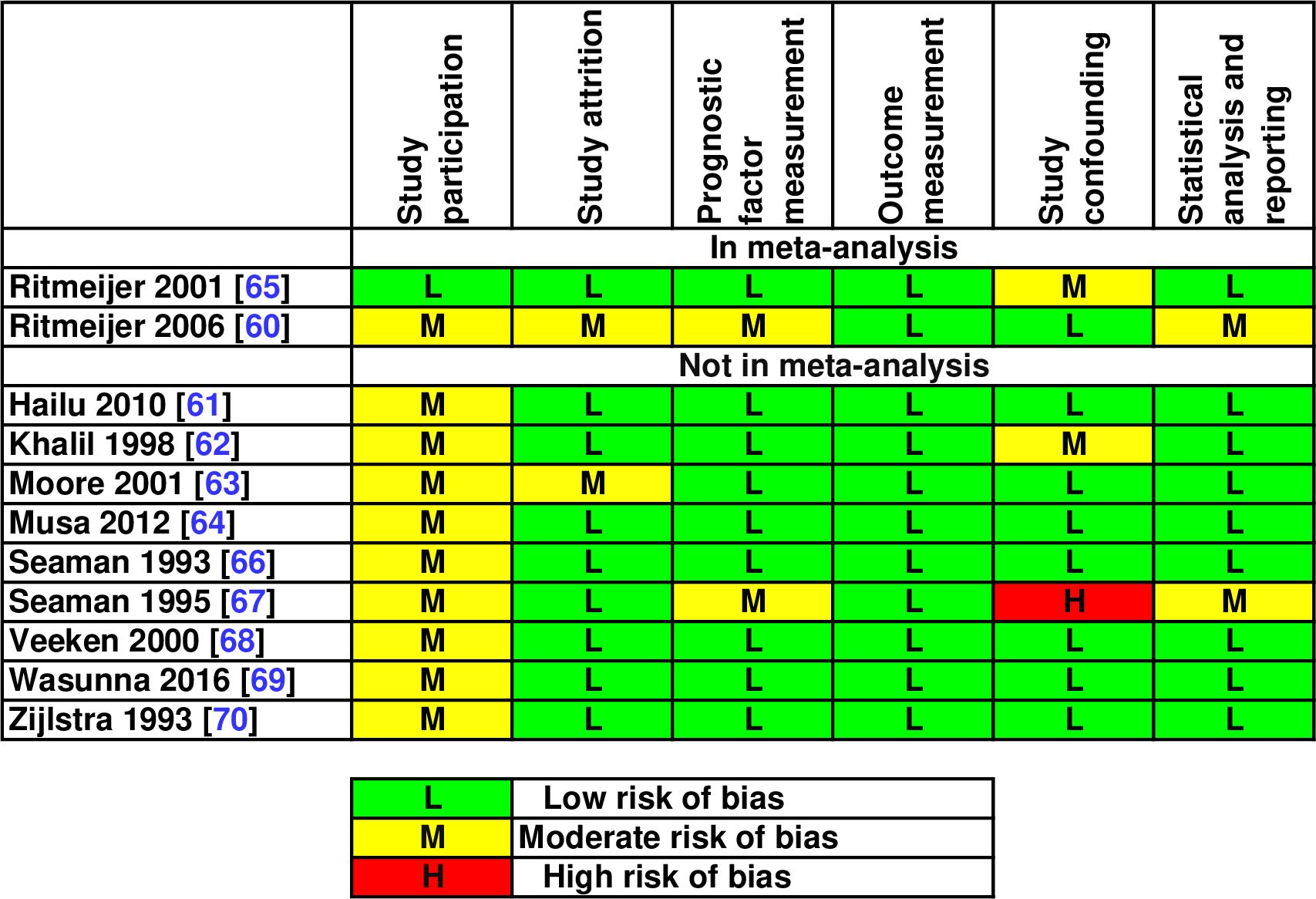

Supplement: S2 Fig — (Figure structure: on top “studies included in the meta-analysis”–below: “studies included in narrative synthesis only”). (JPG) [file pntd.0008319.s006.jpg]
